# Comparison of ultrasound-guided supraclavicular and costoclavicular brachial plexus block using a modified double-injection technique: a randomized non-inferiority trial

**DOI:** 10.1042/BSR20200084

**Published:** 2020-06-09

**Authors:** Quehua Luo, Weifeng Yao, Yunfei Chai, Lu Chang, Hui Yao, Jiani Liang, Ning Hao, Song Guo, HaiHua Shu

**Affiliations:** 1 The Second School of Clinical Medicine, Southern Medical University, Guangzhou, China; 2 Department of Anesthesiology, Guangdong Second Provincial General Hospital, Guangzhou, China; 3 Department of Anesthesiology, The Third Affiliated Hospital of Sun Yat-Sen University, Guangzhou, China; 4 Department of Anesthesiology, Cardiovascular Institute of Guangdong Province, Guangdong Provincial People’s Hospital, Guangdong Academy of Medical Sciences, Guangzhou, Guangdong, China

**Keywords:** brachial plexus block, costoclavicular, double-injection technique, nerve stimulator, supraclavicular, ultrasound

## Abstract

Ultrasound-guided costoclavicular block (CC-approach) is a recently described brachial plexus block (BPB) and an alternative approach to the supraclavicular approach (SC-approach). The relevant sonoanatomy is analogous in terms of the brachial plexus and its adjacent artery for both approaches. In the present study, we hypothesized that the two approaches will result in similar block dynamics when used the modified double-injection (MDI) technique. One hundred and twelve patients were randomly allocated to receive either a SC- or CC-approach with MDI technique. In the CC group, half the volume was injected adjacent to the medial cord of the brachial plexus, the procedure was guided by ultrasound and verified by nerve stimulator, subsequently the second half was injected close to the lateral cord. In the SC group, the MDI technique was carried out as described in our previous study. Sensory and motor blockade of all four terminal nerves were assessed with a 3-point scale. The primary outcome was the proportion of complete sensory blockade at 15 min with a predefined non-inferiority margin of −13%. The proportion of subjects at 15 min was comparable between the SC group and the CC group (91 vs 87%, absolute difference: −3%). No significant differences were found for complete motor blockade and onset times of the individual nerves within 30 min, and block-related serious adverse events (all *P*>0.05). We conclude that the MDI technique applied to a costoclavicular and supraclavicular block resulted in similar block dynamics. In addition, it may provide a promising alternative technique when considering the use of multipoint injection.

## Introduction

Ultrasound-guided supraclavicular (SC) and infraclavicular (IC) approaches have become increasingly common brachial plexus blocks (BPBs) for upper-extremity surgery, because of the greater safety of these methods due to real-time ultrasound guidance and faster onset times [1,2]. We recently proposed that using ultrasound-guided modified double-injection (MDI) technique for the SC-approach could obtain better sensory-motor blockade [3]. The first injection of this technique precisely distributes local anesthetics (LA) to the ulnar nerve (UN) that is originated from the inferior trunk of the brachial plexus by ultrasound-neurostimulation. Then the second placement of LA is located at the center of the main neural cluster with real-time ultrasound guidance, which is floated superficially and clearly with the help of the former injection. As a result, this MDI technique will contribute to ensure the end point of the first injection and the desired pattern of spread of LA is completed following a second injection.

Similarly, the costoclavicular approach (CC-approach), pioneered by Karmakar et al. [4], and performed at the costoclavicular space (CCS), targets the center of the three neural cords lateral to the axillary artery, and has been reported to produce rapid onset times [5,6]. Although the two approaches require injection of LA close to the contiguous compartments of the brachial plexus, the relevant sonoanatomy for both approaches shares a similar connection between the brachial plexus and artery. For the SC-approach, the trunks and divisions of the brachial plexus arrange compactly superolateral to the subclavian artery at the supraclavicular fossa [7,8]. Alternately, at the CCS, all three cords of the brachial plexus are clustered together lateral to the axillary artery [9,10]. The anatomic relationship of the three cords has been found to be relatively constant arrangement, in which the medial cord is located at the innermost level. Usually, the UN is also the largest branch derived entirely from the medial cord. Whereas, studies typically focus on single-injection to obtain satisfactory results until now. Therefore, we assumed that the ultrasound-guided MDI technique applied to the two approaches might result in similar block dynamics, including the proportion of complete sensory or motor blockade and onset times.

Given that the MDI technique has been associated with better block dynamics such as faster onset times, better success rates and complete block for ultrasound-guided a supraclavicular block. The present study examines the block dynamics and clinical usefulness of the MDI technique at the IC level. We hypothesized that the MDI technique can help localize the brachial plexus accurately and guide needle advancement to the target nerves for ultrasound-guided a costoclavicular and supraclavicular block, which would result in comparable proportion of the complete sensory blockade at 15 min after LA injection. Consequently, we designed this randomized study as a non-inferiority trial.

## Materials and methods

### Design and patients

**Figure 1:**
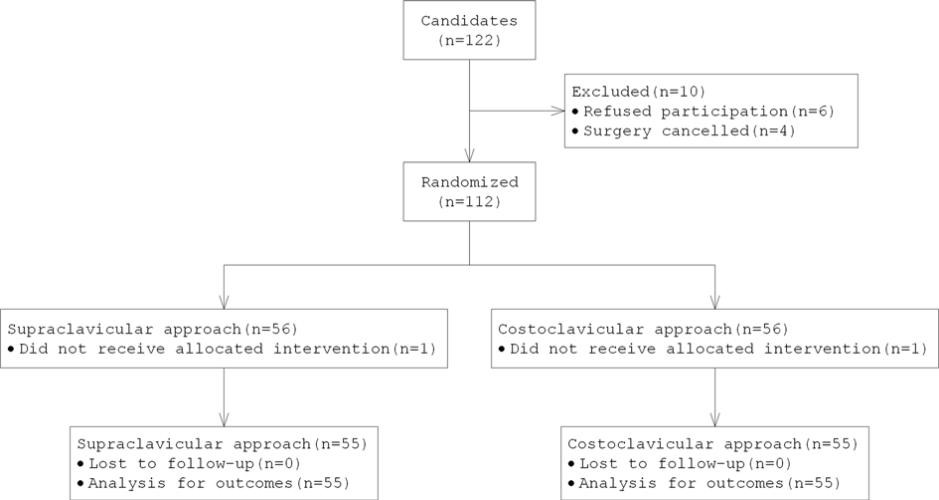
Flow diagram of the present study The present study was designed and conducted on the basis of the Consolidated Standards of Reporting Trials guidelines.

This non-inferiority trial was approved by the local research ethics committee of our hospital (Ethics committee of Guangdong Second Provincial General Hospital) and registered at http://www.chictr.org.cn with the registration number ChiCTR1800018736. The clinical study was conducted at the Guangdong Second Provincial General Hospital from October 2018 to March 2019. Written informed consents were obtained from all candidates. The present study included 112 patients of American Society of Anesthesiologists (ASA) physical status I–III, aged 18–75 years, who were scheduled to undergo surgery of the elbow, forearm, wrist, or hand with BPB (Figure 1). Exclusion criteria included the following: patient’s refusal, coagulopathy, pre-existing neuropathy, infection at the supraclavicular or IC fossa, allergy to LAs, pregnancy and body mass index more than 30 kg/m^2^.

### Randomization and blinding method

Patients were randomly allocated to one of the two study groups: supraclavicular or costoclavicular block. The drawing sequentially numbered, sealed, opaque envelopes were prepared, and they contained a card with the computer-generated allocation number:1 = SC-approach, 2 = CC-approach. The envelopes were prepared and announced by a research assistant who no longer engaged in further study. Outcome assessors were blinded to the randomization sequence and in charge of evaluating and recording clinical data. The observers who were also blinded to the used technique performed the follow-up at 72 h postoperatively.

### Ultrasound-guided techniques

On arrival to operating room, standard monitors (non-invasive cuff blood pressure, pulse oxygen saturation, and electrocardiogram) and supplemental oxygen (nasal cannula at 4 l/min) were applied. All patients received intravenous premedication (midazolam 0.05 mg/kg) when venous access (20-gauge) was established. For both the approaches, a portable ultrasound machine (Sonosite M-turbo, SonoSite, Inc., Bothell, WA) with a 6-13 MHz linear array transducer (38-mm footprint) and a 22-gauge, 50-mm, short-beveled stimulating needle (B. Braun Melsungen AG, Melsungen, Germany) were used for all participants. All blocks were performed by experienced anesthesiologists who have had extensive experience with ultrasound-guided SC- or CC-approach before the research.

**Figure 2:**
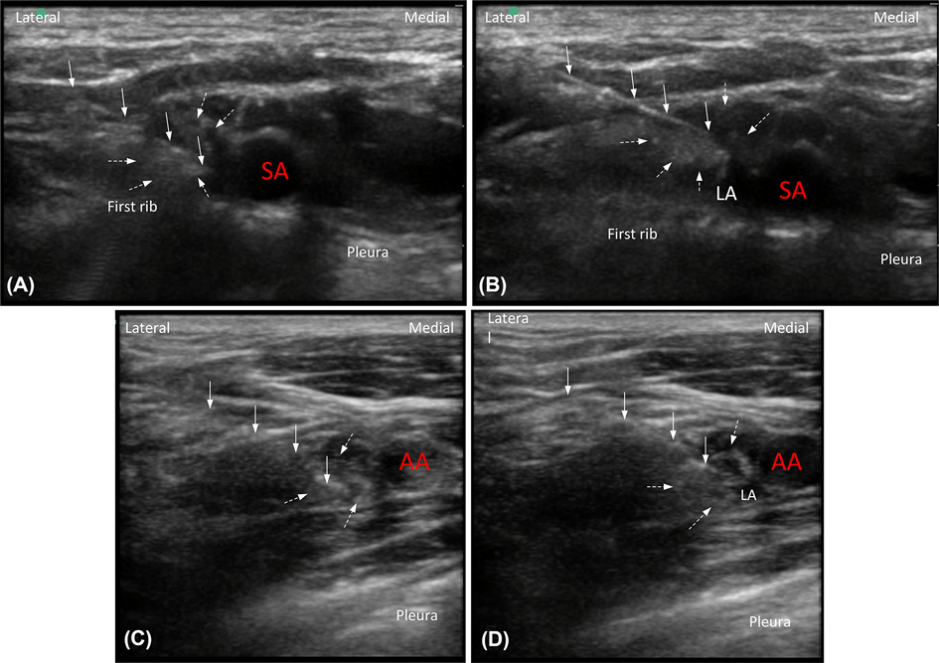
Image demonstrating the MDI technique used at the supraclavicular fossa and the costoclavicular space Transverse sonogram showing the images of the supraclavicular block, in which the trunks and divisions of the brachial plexus can be visualized as hypoechoic circular structures (dotted arrows), lateral to the SA at the supraclavicular fossa: (**A**) First step: the needle tip (solid arrows) was orientated to the junction of the first rib and subclavian artery (called ‘corner pocket’); the LA injection targeted the desired responses of the areas innervated by UN. (**B**) Second step: LA was administered into the center of the main neural cluster. And transverse sonogram showing the images of the costoclavicular block, in which the three cords of the brachial plexus can be visualized as hypoechoic circular structures (dotted arrows) lateral to the AA at the costoclavicular space: (**C**) First step: the needle tip (solid arrows) was orientated to the medial cord of the brachial plexus lateral to the AA, and verified with a peripheral nerve stimulator. (**D**) Second step: LA was administered close to the lateral cord with real-time ultrasound guidance. Abbreviations: AA, axillary artery; SA, subclavian artery.

The ultrasound-guided SC block with a MDI technique was performed following the procedure described in our previous study [3]. The first step was to initiate an ultrasound scan for a satisfactory image, which was a short-axis view of the supraclavicular fossa and elliptical hypoechoic trunks and divisions arranged compactly superolateral to the subclavian artery. The anesthesia provider initially oriented the needle tip to the target location, called the ‘corner pocket’, using an in-plane technique in lateral to medial direction, while the electric current was turned on to 0.4 mA (frequency: 2 Hz, pulse width: 0.1 ms) during the procedure (Figure 2A). The purpose of the peripheral nerve stimulator was to elicit the sensory or muscle twitch responses of the areas innervated by the UN: flexion or paresthesia of the fourth and the fifth finger or thumb adduction. Half the volume (11.5 ml) of a 1:1 mixture of 2% lidocaine and 1% ropivacaine was injected after the accurate position was confirmed by ultrasound-neurostimulation. Subsequently, the remaining volume (11.5 ml) was administered into the center of the main neural cluster formed by the trunks and divisions (Figure 2B).

For the CC-approach, nerve blocks were performed at the level of the cords as previously described [4]. The patients were placed in a supine position with the arm abducted at 90 degrees. When initially scanning from the midpoint of the clavicle toward the CCS, the three cords (lateral, medial, and posterior) could be visualized and were clustered tightly lateral to the axillary artery. The needle was advanced from the lateral end of the probe, in plane with the ultrasound beam, as the electric current was set at 0.4 mA (2 Hz and 0.1 ms). The same type and volume (11.5 ml) of LA was first injected to the targeted nerves when the desired sensory-motor responses of the areas innervated by the medial cord (finger flexion) or UN (which derives entirely from the medial cord of the brachial plexus) were elicited as the aforementioned response (Figure 2C). Subsequently, the remaining volume (11.5 ml) was injected to the lateral cord under closely observation with real-time ultrasound guidance (Figure 2D).

### Outcome measures

The primary outcome was the proportion of complete sensory blockade at 15 min after LA injection. The sensory blockade of the four nerves was evaluated and graded every 3 min until 30 min after injection by double-blinded observers using a 3-point scale (0 = no block, 1 = partial anesthesia, 2 = complete anesthesia). Similarly, motor block was tested and graded according to a 3-point scale: 0, no block; 1, paresis; 2, paralysis. The overall sensory and motor scores were calculated for every patient at the predetermined intervals. For purposes of standardization, the complete sensory or motor blockade were defined as score was equal to or greater than 7 points. The onset times were defined as the measured interval when a complete sensory or motor blockade was achieved. Sensory blockade was evaluated in the cutaneous distribution of each nerve using a cold test (ice) on the following: the lateral forearm for the musculocutaneous nerve (MCN), the palmar aspect of the second finger for the median nerve (MN), the dorsum of the hand between the thumb and second finger for the radial nerve (RN), and the ventral side of the fifth finger for the UN. Motor blockade of each nerve was evaluated by elbow flexion (MCN), wrist flexion and opposition of the second and third fingers and the thumb (MN), wrist extension (RN), and flexion and opposition of the fifth finger toward the thumb (UN).

The secondary outcomes included the performance time, satisfactory imaging, number of needle passes, procedural-related pain, difficulty level, needle visual score, and adverse events. For both techniques, the performance time was defined as the time interval between contact of the ultrasound probe with the skin and removal of the needle. Satisfactory imaging was observed in real time and must meet the criteria that two of the three trunks of the brachial plexus should be visualized as hypoechoic circular structures lateral to the subclavian artery for the SC-approach; and the three cords should be visualized and clustered tightly lateral to the axillary artery in a single ultrasound window for the CC-approach. An additional needle pass was defined as at least 10-mm withdrawal of the needle to retract its trajectory. Procedural-related pain was assessed with the numeric rating scale (NRS): 0, no pain; 10, worst possible pain. Needle visual score was evaluated using a 5-point scale: 1 = very poor, 2 = poor, 3 = fair, 4 = good, 5 = very good. Assessment was made at that time when the first proper position for the LA injection was confirmed and the visibility was theoretically optimized. The incidence of vascular puncture, Horner syndrome, toxicity of LA, pneumothorax, and difficult level (0, none; 1, mild; 2, moderate; 3, severe) were also documented. In addition, patients routinely received a dexmedetomidine (0.2–0.7 µg/kg per hour) infusion in preventing of anxiety during surgery, provided response to verbal stimulus was maintained. Once a complaint of pain was made during surgery, the block was considered a failure, and patients were allowed to receive remedial measures at the discretion of the responsible anesthesiologist. Suspicious symptoms of nerve injury such as persistent paresthesia or deficits were monitored and assessed at the 72-h follow-up after surgery.

### Sample size calculation and statistical analysis

The present study was designed as a non-inferiority trial. Sample size was based on the primary end point (the proportion of subjects with complete sensory blockade at 15 min). According to a pilot study (*n*=15), the proportion of subjects for ultrasound-guided supraclavicular block with MDI technique was 93.3% (14 out of 15). Thus, we used 93% as the established proportion in both groups, a power of 80%, one-sided 95% confidence interval, and at least 80% of those who received a costoclavicular block was designed as a non-inferiority trial. The required number of cases in each group was calculated to be 48. A total of 112 patients were calculated to account for the anticipated 15% dropout rate.

Statistical analysis was carried out using *SPSS for Windows 18.0* (SPSS Inc, Chicago, Illinois). Categorical variables were summarized as a frequency, *n* (%), and the two study groups were compared using the Pearson *χ*^2^ test, or Fisher’s exact test. Data were presented as mean ± standard deviation (SD) or median and interquartile range depending on the distribution of the data. Normality of the data for continuous variables were assessed using the Kolmogorov–Smirnov test and analyzed using an independent-samples *t* test or a Mann–Whitney U test, as appropriate. *P*<0.05 was considered statistically significant for all results.

## Results

**Figure 3:**
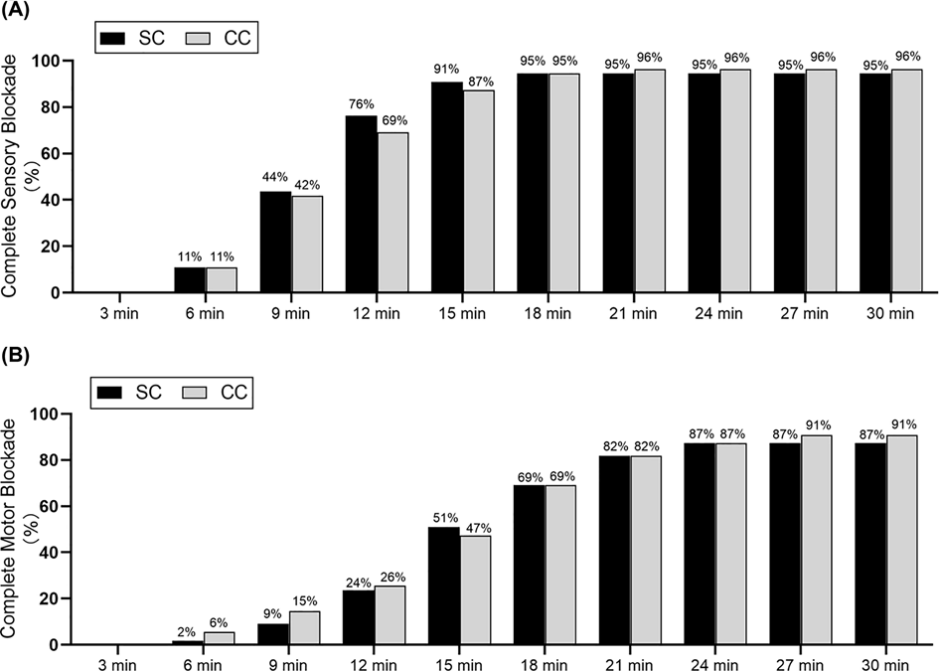
Percentage of patients with complete sensory blockade or motor blockade Sensory blockade (**A**) or motor blockade (**B**) were observed at 3-min intervals. There were no significant difference between the two groups at all the predetermined intervals (all *P>0.05*). Abbreviations: CC, costoclavicular; SC, supraclavicular.

Each group had one patient dropout. No differences in patient characteristics or surgical procedure were observed between the two groups (Table 1). In these two study groups, the proportion of patients with complete sensory, motor blockade at various time points are presented in Figure 3A,B respectively. The proportion of overall sensory blockade at 15 min after injection in the CC group was non-inferior to that in the SC group (87 vs 91%; absolute difference: −3%) (Figure 3A). No significant differences were found for complete sensory, motor blockade at all the predetermined intervals within 30 min (all *P*>0.05) (Figure 3A,B). Similarly, there was no statistically significant difference in the onset times of overall sensory or motor blockade between the two groups (Table 2). The SC group required fewer needle passes and had a shorter performance time (all *P<0.05*). Furthermore, the MDI technique had a relatively poor performance in difficulty degree and needle visualization in the CC group (all *P<0.05*). However, no differences were found in surgical anesthesia, procedural-related pain, and satisfactory imaging (all *P*>0.05). Higher rates of Horner syndrome were seen in the SC group than in the CC group (29% vs zero, *P*<0.01), but there was no difference between the two groups with regard to vascular puncture, toxicity of LA, pneumothorax and operative duration. No neurological deficits were noted at the 72-h follow-up after surgery (Table 3).

**Table 1:** Patient characteristics

	SC group (*n=55*)	CC group (*n=55*)	*P*-value
Age (years)	44.5 ± 14.2	40.3 ± 13.3	0.12
Sex			0.70
Male	31 (56.4%)	29 (52.7%)	
Female	24 (43.6%)	26 (47.3%)	
BMI (kg/m^2^)	22.4 ± 2.5	22.8 ± 3.5	0.48
ASA			0.91
I	27 (49.1%)	25 (45.5%)	
II	23 (41.8%)	24 (43.6%)	
III	5 (9.1%)	6 (10.9%)	
Operative site			0.63
Elbow	13 (23.6%)	9 (16.4%)	
Forearm	17 (30.9%)	19 (34.5%)	
Distal wrist	25 (45.5%)	27 (49.1%)	

Data are presented as mean ± SD or number (%). There were no statistically significant differences identified. Abbreviation: BMI: body mass index.

**Table 2:** The onset times of all four nerves

	SC group (*n=55*)	CC group (*n=55*)	*P*-value
Overall sensory onset time			
UN (min)	6 [6–9]	9 [6–10.5]	0.19
MN (min)	9 [6–12]	9 [6–12]	0.68
RN (min)	12 [9–15]	12 [9–15]	0.45
MCN (min)	12 [9–12]	12 [9–15]	0.45
Overall motor onset time			
UN (min)	12 [9–15]	12 [8.25–12]	0.52
MN (min)	15 [12–18]	15 [12–21]	0.82
RN (min)	15 [12–18]	15 [12–18]	0.93
MCN (min)	15 [9–18]	15 [9–18]	0.71

Data are presented as median [IQR]. There were no statistically significant differences identified. IQR: interquartile range

**Table 3:** Block-related parameters and outcomes

	SC group (*n=55*)	CC group (*n=55*)	*P*-value
Performance time (s)	251.69 ± 43.17	274.55 ± 45.62	0.01
Surgical anesthesia (yes/no)	54/1	53/2	0.56
Satisfactory imaging (yes/no)	39/16	34/21	0.31
Number of needle passes (number)	4 [3–5]	5 [4–5]	<0.01
Procedural-related pain (0–10)	2 [1–3]	2 [1–3]	0.24
Difficult degree (0–3)	1 [1–2]	2 [1–2]	<0.01
Needle visual score (1–5)	4 [3–4]	3 [3–4]	<0.001
Horner syndrome (yes/no)	16/39	0/55	<0.01
Vascular puncture (yes/no)	1/54	2/53	0.56
Toxicity of LA (yes/no)	0/55	0/55	-
Pneumothorax (yes/no)	0/55	0/55	-
Nerve injury (yes/no)	0/55	0/55	-
Operative duration (min)	133.8 ± 40.3	125.3 ± 37.3	0.254

Continuous variables are presented as mean ± SD. Categorical variables are presented as number. Ordinal variables (number of needle passes, procedural-related pain, difficult degree, and needle visual score) are presented as median [IQR].

## Discussion

The results of this double-blinded randomized study suggest that in terms of complete sensory, motor blockade and onset times, a costoclavicular BPB shares similar block dynamics with a supraclavicular block. Both approaches had similar proportion of complete sensory blockade at 15 min (87 vs 91%; absolute difference: −3%), the difference which falls within our *a priori* accepted non-inferiority margin (−13%). Despite the difference in needle trajectory, the percentage of patients that had complete sensory and motor blockade with a 7-point minimal composite score at 15 and 30 min for the CC-approach echo the findings for the SC-approach (47 vs 51%; 91 and 87%, respectively). Compared with the SC-approach, it is noteworthy that the MDI technique required more needle passes for the CC-approach, but the additional needle redirections did not lead to an increased incidence in pneumothorax, vascular puncture, or postoperative nerve injury. Hence, the MDI technique also proved to be a useful method with a satisfying sensorimotor blockade when applied to a costoclavicular block.

On the basis of the similar anatomical arrangement of the brachial plexus at the CCS compared with that at the supraclavicular fossa, the MDI technique might also have good application value at the IC-BPB. The single-injection technique used commonly for the CC-approach, with or without a peripheral nerve stimulator, has a high success rate (97%) that might be partially explained by the large LA volume used for the procedure (up to 35 ml) [6,9]. Recently, a letter has described the double injection technique for CC-approach, and the described two-injection technique resulted in high success rate of successful anesthesia (97.5%) [11]. The MDI technique used in this study is similar to that letter reported. The essence of the MDI technique can be simplified as first targets the most desired part of the brachial plexus, and then the remaining parts are fully exposed. For MDI technique, it is the preferred targeting the medial cord, and second for lateral cord in our study. Based on cadaver studies, fascial layers are usually found (up to 60%) around the neurovascular sheath [10,12,13]. In addition, according to the report by Sala-Blanch et al. [10], the brachial plexus was very compact at this level, and made up of three cords with the medial cord located at the innermost level. The existence of fascial layers around the neurovascular bundle had been found to separate the lateral cord from the other two cords (medial and posterior) at the CCS. Expectedly, the MDI technique produced the fastest onset time of sensory and motor blockade of the UN than for the other three nerves. The onset time of MCN blockade (sensory onset time 12 [IQR, 9–15] min; motor onset time 15 [IQR, 9–18] min) was satisfactory for the CC-approach as well, compared with the previous research data [9]. Furthermore, imaging difficulties due to short, wide necks are occasionally encountered in SC-approach, but the CC-approach should be feasible in almost all patients theoretically. It also has the anatomical advantages of both SC-approach and axillary approach: a similar sonoanatomy between the brachial plexus and artery allowing single or multipoint injection of LA and a diminished risk of pneumothorax. Therefore, we do recommend the CC-approach as an alternative approach if unsatisfactory ultrasound imaging of the supraclavicular fossa (for example, because of a short neck) makes it unsafe and *vice versa*.

Despite the introduction of multipoint injection for extended blockade and shorter onset times, those techniques also present some confusion [1,14]: (i) the preferred site of injecting the first volume of LA for multipoint injection, especially the double-injection technique, (ii) the added value of peripheral nerve stimulation combined with ultrasound guidance, and (iii) the optimal volume of LA is used due to visual technology. Some studies have shown that, compared with ultrasound alone, combined ultrasound-neurostimulation resulted in comparable success rates and total anesthesia-related times, as the shorter onset time was counterbalanced by its longer performance time for the SC- or IC-BPB [3,15,16]. However, due to improvement of knowledge in anatomy and proficiency in clinical practice, these permit the achievement of a shorter total anesthesia time for nerve block in hidden deep tissue (lumbar plexus block) [17]. Thus, we assume that nerve stimulation will have no benefits for superficial nerve blocks such as BPB, just in terms of total anesthesia time but not in terms of the success rate and onset times at the predetermined intervals [17,18]. In this study, we achieved a relatively satisfying success rate at various time points for the CC-approach. These results outdid the success rate in similar studies. This single-injection method was frequently associated with incomplete blockade of the medial or lateral cords of the brachial plexus [5,9]. We are starting to believe that neurostimulation is not merely used to shorten onset time. Instead, it may serve as a defense mechanism for identifying needle endpoint during seeking the first injection site.

In the present study, a costoclavicular block was associated with more needle passes than a supraclavicular block, and this most likely because of bony encounters, needle direction and poor visualization. In addition, self-assessment of difficulty degree also presented greater challenges for experienced anesthesiologists. These are consistent with the results came from previous study [19,20]. According to Nieuwveld et al. study, a medial to lateral approach instead of the lateral to medial was proposed to solve the aforementioned problems [20]. However, procedural-related pain did not correlate directly with multiple needle passes. This discrepancy may be partially explained by the higher percentage of non-neural tissue at CCS compared with that at supraclavicular fossa [13]. On the other hand, this could be due to the fact that sedation was administrated to all patients and the puncture position was distal from the neck. Previously, we chose a relatively small LA volume (23 ml) for the SC-approach since it represented the documented minimum effective anesthetic volume in 50% of subjects [21], and now allowed us to compare the outcome under the same condition. In previous studies, 30–90% of subjects experienced Horner syndrome when a high volume of LA (30–35 ml) used for supraclavicular blocks [22,23], Patients were subjectively felt some uncomfortable about the temporary nature of the phenomenon, oxygen therapy and continuous observation but no additional treatment was required. In the SC group, 29% of the participants experienced Horner syndrome, this incidence falls within the abovementioned range, whereas the incidence of this complication was zero in the CC group. This may be due to the fact that nerves at the costoclavicular space are further away from the trajectory of cervical sympathetic nerve and the limited diffusion capacity of a relatively small volume of LA.

Our protocol does have some limitations. First, we chose to perform the MDI technique with two different approaches at the contiguous segments of the brachial plexus. We favored the ultrasound-neurostimulation technique for a supraclavicular block in daily clinical practice and employed this technique in a costoclavicular block based on similar anatomical characteristics and ultrasound image. The benefits of the traditional double-injection with ultrasound only in pursuing faster complete sensory and motor blockade or onset times for a costoclavicular block are unclear. Randomized controlled trials may be needed to assess individual safety and efficiency between the classical and MDI techniques and if the clinical feasibility of the MDI technique is valuable for continuous catheter analgesia or teaching. Second, we used a total volume of 23 ml for both approaches, which was described in our previous study. This volume had demonstrated that a perfectly adequate injection volume was able to anesthetize the brachial plexus. Moving forward, volume-finding study exploring an optimal injectate for both approaches with the MDI technique, would help control the bias. Third, the safety of needletip placement inside the neural clusters is controversial. Most studies seem to support the safety and effectiveness of subfascial injection technique [23–25,15], however a recent cadaver study reported that a single ultrasound-guided intracluster injection could lead to a high risk of sub-perineural injections (24%) in the supraclavicular fossa [26]. A stimulating current of 0.4 mA was commonly used to avoid intraneural injection and we did discontinue needle advance and LA injection in the presence of pain or high injection resistance in the present study. Nonetheless, further studies are needed to verify the safety of multipoint targeted intracluster injection.

In conclusion, ultrasound-guided costoclavicular and supraclavicular block resulted in similar block dynamics with the MDI technique. There were no serious complications directly related to the technique or LA injection. In addition, the MDI technique resulted in satisfying success rates of all four nerves, therefore it may provide a promising alternative technique when considering the use of multipoint injection (≥2 sites).

## Abbreviations

BPB: brachial plexus block
CC-approach: costoclavicular block
CCS: costoclavicular space
IC: infraclavicular
LA: local anesthetics
MCN: musculocutaneous nerve
MDI: modified double-injection
MN: median nerve
RN: radial nerve
SC-approach: supraclavicular approach
SC: supraclavicular
UN: ulnar nerve
